# Effect of ACADL on the differentiation of goat subcutaneous adipocyte

**DOI:** 10.5713/ab.22.0308

**Published:** 2023-01-11

**Authors:** A Li, YY Li, QB Wuqie, X Li, H Zhang, Y Wang, YL Wang, JJ Zhu, YQ Lin

**Affiliations:** 1 Key Laboratory of Qinghai-Tibetan Plateau Animal Genetic Resource Reservation and Utilization of Education Ministry, Southwest Minzu University, Chengdu 610041, China; 2 Key Laboratory of Qinghai-Tibetan Plateau Animal Genetic Resource Reservation and Exploitation of Sichuan Province, Southwest Minzu University, Chengdu 610041, China; 3 College of Animal and Veterinary Science, Southwest Minzu University, Chengdu 610041, China

**Keywords:** Acyl-CoA Dehydrogenase Long Chain (ACADL), Adipocytes, Differentiation, Goat, Subcutaneous

## Abstract

**Objective:**

The aim of this study was to clone the mRNA sequence of the Acyl-CoA dehydrogenase long chain (*ACADL*) gene of goats and explore the effect of ACADL on the differentiation of subcutaneous fat cells on this basis.

**Methods:**

We obtained the *ACADL* gene of goats by cloning and used quantitative real-time polymerase chain reaction (qPCR) to detect the ACADL expression patterns of different goat tissues and subcutaneous fat cells at different lipid induction stages. In addition, we transfect intramuscular and subcutaneous adipocytes separately by constructing overexpressed ACADL vectors and synthesizing Si-ACADL; finally, we observed the changes in oil red stained cell levels under the microscope, and qPCR detected changes in mRNA levels.

**Results:**

The results showed goat *ACADL* gene expressed in sebum fat. During adipocyte differentiation, ACADL gradually increased from 0 to 24 h of culture, and decreased. Overexpression of ACADL promoted differentiation of subcutaneous adipocytes in goat and inhibited their differentiation after interference.

**Conclusion:**

So, we infer ACADL may have an important role in positive regulating the differentiation process in goat subcutaneous adipocytes. This study will provide basic data for further study of the role of ACADL in goat subcutaneous adipocyte differentiation and lays the foundation for final elucidating of its molecular mechanisms in regulating subcutaneous fat deposition in goats.

## INTRODUCTION

Compared with other meats, goat meat has high nutritional value, so people pay great attention to the characteristics of goat meat, including the color, marbling, tenderness, and flavor [1]. Subcutaneous fat is an important factor, which affects meat quality and influences the number and volume of adipocytes. During early development in goats, adipose deposition is mainly caused by adipocyte formation and differentiation, subcutaneous fat deposition affected by numerous factors. The regulatory role of numerous genes plays an important role in the differentiation of adipocytes [2,3]. However, the gene regulatory networks involved have not been fully studied. Therefore, it is of great significance to further explore the molecular mechanism of subcutaneous fat deposition from the genetic level to improve meat quality.

Long-chain acyl coenzyme A dehydrogenase (ACADL) is the first step in catalytic fatty acid oxidation and plays an important role in long-chain fatty acid oxidation including expression regulation and activity regulation. ACADL is a key factor in multiple metabolism pathways, and over-expression of ACADL enhanced secretion of interleukin-6 (IL-6) and IL-10 in macrophages [4]. It has been shown that ACADL has been implicated in malignant tumor growth [5]. Sometimes it’s deficiency or absence can also cause mitochondrial dysfunction [6,7]. ACADL is not only a key protein in the liver metabolism, but also an important regulator in lipid metabolism [8,9]. Based on previous reports, ACADL expression is associated with fat deposition, however, the effects of ACADL on subcutaneous adipocytes have not been studied in goats.

Therefore, the goat *ACADL* gene was cloned in this study and bioinformatics was analyzed. In addition, quantitative real-time polymerase chain reaction (qPCR) was used to detect the expression patterns of *ACADL* in various tissues and subcutaneous adipocytes at different stages of differentiation. Subsequently, oil red O staining and qPCR, respectively, enabled the study the goat subcutaneous preadipocyte transfection with ACADL overexpression plasmid and si-ACADL explored its function at the morphological and molecular levels. This study lays a foundation for future research on the differentiation of subcutaneous adipocyte with ACADL.

## MATERIALS AND METHODS

### *ACADL* gene cloning and bioinformatics analysis

The experiments in this study met the requirements of the “List of Ethical Treatment of Laboratory Animals in China” complied with the requirements of the directory of the Ethical Treatment of Experimental Animals of China. All animal experiments were reviewed by Animal Experimental Ethical Inspection of Southwest University for Nationalities (No. 10832). In this experiment, five healthy male goats purchased from Jian Zhou Da-er goat were selected for the experiment and tissue samples were collected from heart, liver, spleen, lung, subcutaneous fat and muscles (longest dorsal muscle, femoral biceps, and arm triceps of the arm). We isolated total RNA according to the instructions of trizol kit (Takara, Dalian, China). RNA concentration and mass were then measured using IMPLEN NanoPhotometer N60. The first strand of cDNA was synthesized by the Revert Aid First Strand cDNA Synthesis Kit (Thermo, Waltham, MA, USA). Primers were designed by Primer 5.0 according to GenBank (XM_018059958). Primer information is shown in Table 1. The total PCR system is 25 μL, consisting of primer STAR MAX primes (2×)12.5 μL, the sense primer and antisense primers 1 μL each, the template(liver) cDNA 1 μg, and the ddH2O 9.5 μL. PCR procedure, 98°C 3 min, 60°C 10 s, 72°C 15 s 35 cycle, 72°C 5 min. PCR products were detected by 1% agarose gel electrophoresis. The Mini Kit for DNA Extraction with Fast pure Gel DNA (Vazyme, Nanjing, China) was used to recover and purify the DNA, and then attached to the 007VS vector (TSINGKE, Chengdu, China), converted to the trelief^TM5a^, positive colonies were picked up on Amp+ plates and identified by PCR, and then sent to Songon Biotechnology Co, Ltd. (Chengdu, China) for sequence analysis. The tools for bioinformatics analysis of goat ACADL are shown in Table 2.

### Cell culture

Preadipocytes were obtained from 7-day-old Jian Zhou Da-er goat (n = 5). First, subcutaneous fat adipose tissue was sampled, washed in phosphate-buffered saline (PBS) supplemented with 1% penicillin/streptomycin, and then chopped under sterile conditions. Enzymatic digestion was performed with 0.2% type II collagenase (Sigma, Tokyo, Japan) at 37°C and terminated with equal volumes of DMEM/F12 (Hyclone, Logan, UT, USA) with 10% fetal bovine serum (Gemini, Calabasas, CA, USA). Secondly, digestion solution was removed and added to 2 mL culture medium. After cell passage third (F3), the cells are used for experimental treatment.

### The construction of ACADL overexpression plasmid and interference sequence synthesis

Using primer 5.0 software to design subcloning primers ACADL-S (CATGCTAGCAGGCCACGCGCCTCCT, CAT CTAGC is the restriction site of NEHI, and start codon is the ATG) and ACADL-A (CGCGGATCCCGGGATGTGG GCAGATGTCTAC, CCGCGGATCC is restriction site of BAMHI, stop codon is the TAG) Goat ACADL plasmid was used as template to amplify it’s coding domain sequence (CDS). Then we used PCDNA3.1 as vector. Second digestion was performed with CDS vector by BamHI (Thermo, USA) and NEHI (Thermo, USA) 37°C 30 min, then purifying and ligating with T4 ligase (Takara, China) in 16°C water bath 16 h. Last the recombinant vector was identified by enzyme digestion and sequencing. The goat ACADL interference sequence and NC (negative control) sequence were synthesized by Shanghai Gene pharma Bio Company (Shanghai, China). ACADL-siRNA sense primer: GCCUGUACAAUU UGAAUAUTT, antisense primer: AUAUUCAAAUUGUA CAGGCTT. NC sense primer: UUCUCCGAACGUGUC ACGUTT, antisense primer: ACGUGACACGUUCGGA GAATT.

### Cell transfection

When goat subcutaneous preadipocyte growth is confluent to 80%, we transfect empty pcDNA-3.1 and pcDNA-3.1-ACADL, respectively, and take 1 μg of pcDNA-3.1 and pcDNA-3.1-ACADL plasmids with 4 μL transfection Reagent (Turbofect; Invitrogen, Waltham, MA, USA) and 200 μL OPTI-MEM per 12 well plate, respectively, and incubate the mixture at room temperature for 15 min. Add the mixture to preadipocytes and mix well. Finally, we remove transfection solution after 16 h and use 50 μM/L oleic acid induces preadipocytes differentiation for 48 h.

### Chemical synthesis and transfection of siRNA

siRNA is synthesized by Gene pharma (Shanghai), Subcutaneous preadipocytes were seeded in 12-well plates. Confluence with 80% when subcutaneous preadipocytes grow. According to the transfection kit (Turbofect, Waltham, USA) instructions, cells are collected 48 h after induction of differentiation, and siRNA is extracted.

### Oil red staining

Cultured cells were washed with PBS twice (reaction 5 min each time) and then fixed with 4% formaldehyde for 15 min at room temperature. Cells were stained with oil red working solution for 20 min and the shape of lipid droplets under a microscope observed and an image taken of the droplets. Added 1 mL isopropanol to each well and the oil red dye eluted. Finally, all stained adipocytes were extracted and added to a 96-well plate to quantify the signal by measuring the optical density at 490 nm (OD490).

### Quantitative real time polymerase chain reaction

Ubiquitously expressed transcript (*UXT*) gene was individually selected as an internal reference gene to normalize mRNA levels. qPCR technology was used to detect the relative expression levels of *ACADL* gene in subcutaneous adipocytes with different differentiation times (0, 12, 24, 36, 48, 60, 72, 84, 96, 108, 120 h). Total PCR system 20 μL (premix 2× TBG 10 μL, ddH2O 7 μL, upstream 1 μL, downstream 1 μL and cDNA 1 μL). PCR procedure pre-degeneration 95°C 3 min, denaturation 94°C 10 s annealing 60°C 20 s extension 72°C 30 s with 40 cycles. Marker genes for differentiation (APETALA-2-Like transcription factor gene [*AP2*], CCAT enhancer binding protein [*C/EBPα*], CCAT enhancer binding protein [*C/EBPβ*], lipoprotein lipase [*LPL*], peroxisome proliferator activated receptor gamma [*PPARγ*], preadipocyte factor 1 [*PREF-1*], and sterol regulating element binding protein isoform 1 [*SREBP1*]) were used to detect the effects of over-expression ACADL and SI-ACADL. Primers were preserved by the laboratory as shown in Table 1.

### Statistical analysis

Data processing and analysis using “mean±standard difference” to represent data, and 2^−ΔΔCt^ was used to homogenize the data obtained by qPCR. SPSS18 analytical software was used to perform one-way analysis of variance. At p<0.05, the difference was considered to be statistically significant. In this study all experiments were repeated three times.

## RESULTS

### Gene cloning of goat ACADL and analysis of physiochemical property of protein

To explore the function goat ACADL, through the primers ACADL-S, ACADL-A, we cloned goat *ACADL* gene sequence with 1,541 bp, it contains complete 1,293 bp CDS region with start codon ATG and end codon TGA that coding 430 amino acids (Supplementary Figure S1). ExPASy analysis shows goat ACADL protein formula C_3858_H_6425_N_1293_O_1293_S_242_, and has a predicted molecular weight with 104,602.66 Daltons. In amino acid composition, Ala (alanine) has the highest content about 29.2% (378), Cys (cysteine) with lowest content about 18.7% (242). Significantly, total number of negatively charged residues (Asp+Glu) and positively charged residues (Arg+Lys) equal zero, so goat ACADL protein has no charge. Instability index (II) is compared to be 35.18. Grand average of hydrophilic (GRAVY) was 0.710. Last, we speculate goat ACADL protein maybe a hydrophobic stable protein.

Potential signal peptide analysis showed that there was no signal peptide in ACADL protein and belongs to cytoplasm protein by Signal 4.1 program analysis. ACADL protein of goat has no transmembrane helix, so called non-transmembrane protein, while subcellar localization revealed that the *ACADL* gene of goat mainly located in cytoplasm (22.2%), endoplasmic reticulum (33.3%), and mitochondrial (11.1%) which indicate ACADL plays a biological role.

### Structure analysis of goat ACADL protein

**Figure 1:**
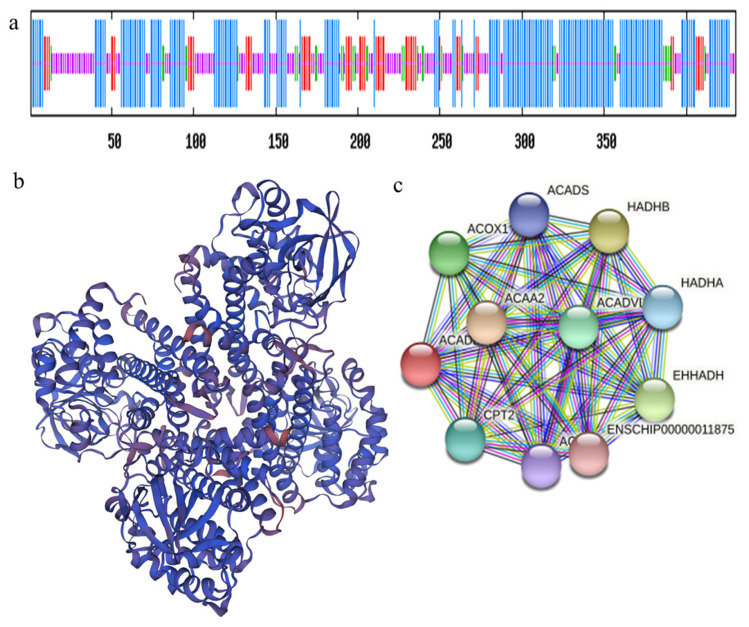
Analysis of protein structure and interaction of goat ACADL. (a) Secondary structure prediction of ACADL protein. Based on the length of vertical from the shortest to the longest, indicating the random coils, beta-turns, extended strands and alpha-helix. (b). ACADL protein three-dimensional structure prediction. (c) Interaction network of ACADL protein. ACADL, Acyl-CoA dehydrogenase long chain.

Online SOPMA server (the prediction of second-level structure) analysis indicated the deduced ACADL protein contained alpha helix207 (48.14%), extended strand 55 (12.79%), beta turn 33 (7.67%), and random coil 135 (31.4%) (Figure 1a). The results of the three-level structure prediction are consistent with the second-level structure prediction (Figure 1b). We used the STRING interactive database to search for proteins interacted with ACADL, the rests suggested that ACADL protein may interact with carnitine palmitoyltransferase-2 (CPT2), Acyl-Co A oxidase 1 (ACOX1), acetyl-coenzyme aly transferase 2 (ACAA2), hydroxyalkyl coenzyme A dehydrogenase A (HADHA), hydroxyalkyl coenzyme A dehydrogenase B (HADHB), very long chain acyl-CoA dehydrogenase (ACADVL) (Figure 1c).

### Goat ACADL homology and phylogenetic tree analysis

**Figure 2:**
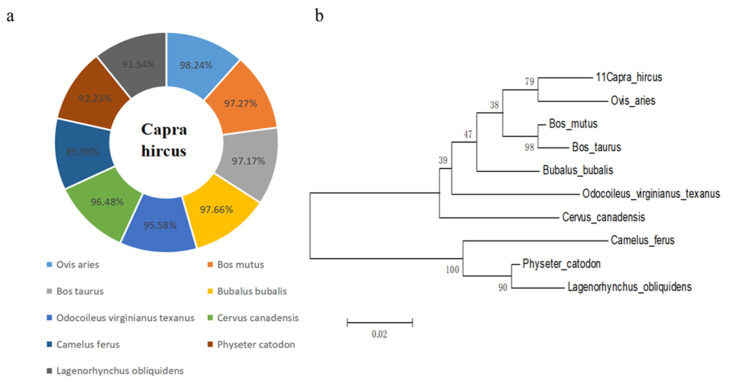
Interspecies comparison analysis and phylogenetic tree construction of ACADL amino acid sequences. (a) Amino acid sequence identity was analyzed by NCBI blast for ACADL protein between goat and other mammalian species retrieved from GenBank. (b) Phylogenetic tree of the goat ACADL. ACADL protein. ACADL, Acyl-CoA dehydrogenase long chain.

**Figure 3:**
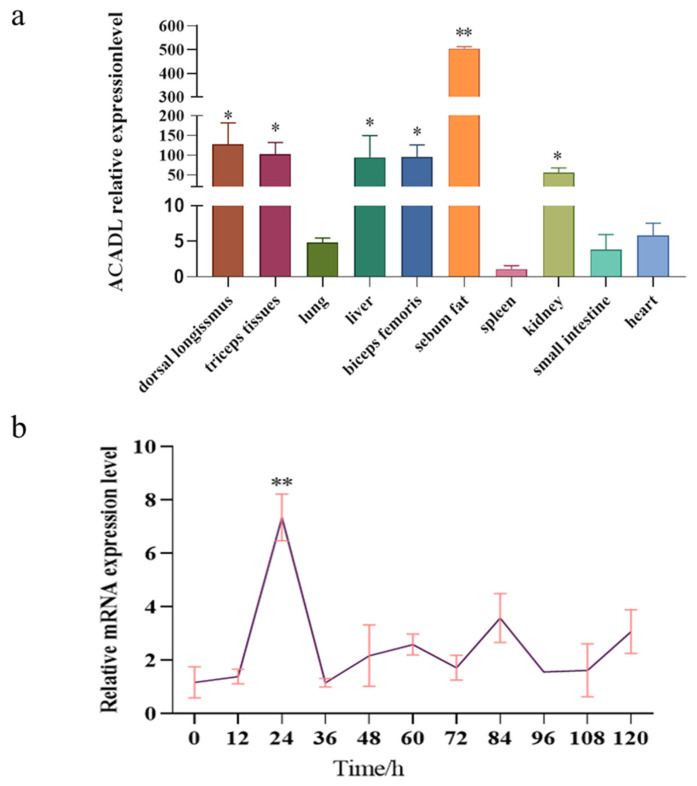
Profile of ACADL tissue expression and temporal expression. (a) Tissue expression profile, relative expression of ACADL in multiple tissues in goat (n = 4), UXT was selected as the internal reference gene to normalize the expression level, “*” means the difference (p<0.05). “**” the difference was extremely significantly compared with other groups (p<0.01). (b) Temporal expression profile, Relative expression level of ACADL during subcutaneous adipocyte differentiation, UXT was selected as the internal reference gene to normalize the expression level, “**” The difference was extremely significant compared with other groups (p<0.01). ACADL protein. ACADL, Acyl-CoA dehydrogenase long chain; UXT, ubiquitously expressed transcript.

Goat ACADL nucleotide shared about 98.24%, 97.27%, 97.17%, 97.66%, 95.58%, 96.48%, 89.98%, 92.23%, 91.54% homology similarity with *Ovis Aries* (XM_027965149.2), *Bos mutus* (XM_005888988.2), *Bos taurus* (XM-024977622.1), *Bubalus bubalis* (XM_006073191.4), *Odocoileus virginianus texanus* (XM_020891105.1), *Cervus canadensis* (XM_0434 45377.1), *Camelus ferus* (XM_032479951.1), *Physeter catodon* (XM_028481521.1), *Lagenorhynchus obliquidens* (XM_027 122437.1) respectively (Figure 2a). A phylogenetic tree was constructed by MEGA5.0 to show the genetic relationship of ACADL in different species (Figure 2b), which was consistent with Figure 3a. Results displayed show that goats were first grouped with Ovis Aries and were the closest relatives, but with the farthest relationship to Lagenorhynchus obliquidens. Goats were clustered with cattle ruminants in the same branch, which is in line with the evolutionary relatedness of these species.

### The tissue and temporal expression profile of *ACADL* gene in goat

We used UXT as the reference gene in this study, ACADL mRNA level was detected in different goat tissues. Our experiment is controlled by heart tissue, and the results show that the *ACADL* gene is widely expressed in goat, while the expression in sebum is significantly higher than in other tissues and lowest in the spleen (Figure 3a). In addition, the dorsal long muscles, triceps, liver, biceps, and kidneys are all higher than other tissues. All the above indicate that ACADL has a high level of expression in sebum and that ACADL is specificity in different goat tissues. To explore the role of *ACADL* gene expression in subcutaneous adipocyte differentiation qPCR was used to detect the expression pattern of *ACADL* gene in the adipocytes that was induced to differentiation from 0 h to 120 h by oleic acid. As shown in Figure 3, ACADL expression gradually increased from 0 h to 24 h, the peak was reached at 24 h, then decreased (Figure 3b). These indicated that ACADL may be a regulator of adipocyte differentiation in goat.

### Over-expression of ACADL on subcutaneous adipocyte

**Figure 4:**
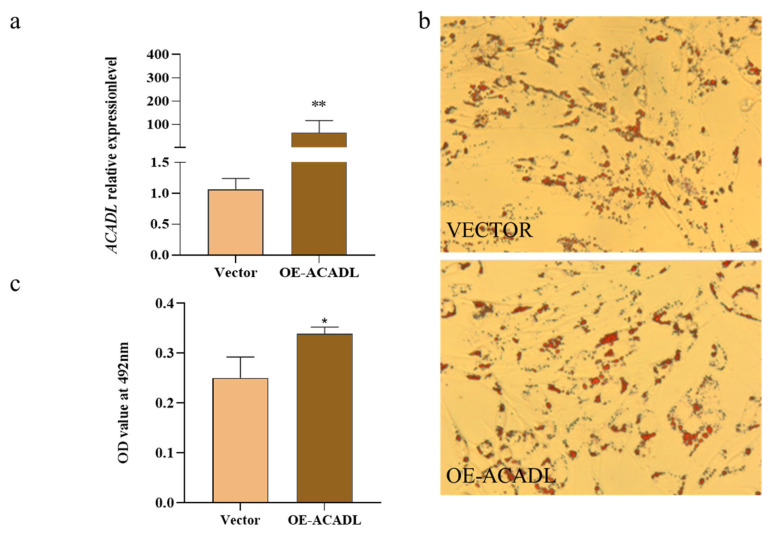
Over-expression of goat ACADL promotes subcutaneous fat differentiation. (a) mRNA expression of ACADL was detected by qPCR after over-expressing ACADL for 48h subcutaneous adipocyte differentiation. (b) Photos of oil red staining of the cells in the tests group (up) and NC group (down) during subcutaneous adipocyte differentiation. (c) The OD value of oil red staining at 492 nm during subcutaneous fat cells differentiation. “**” the difference was extremely significantly compared with control group (p<0.01). “*” The difference was significant (p<0.05). ACADL, Acyl-CoA dehydrogenase long chain.

The effect of ACADL overexpression on subcutaneous adipocyte differentiation in goats, based on data compared to the control group, was elucidated by the qPCR technique. We found that ACADL overexpression significantly upregulated its mRNA levels compared to the control group (Figure 4a). Oil red staining showed that subcutaneous adipocytes after overexpression of the *ACADL* gene were more rounded than controls, secreted more lipid droplets (red part) and had higher OD values (Figures 4b and 4c). In summary, the *ACADL* gene promotes subcutaneous adipocyte differentiation and lipid aggregation.

### Effect of interference ACADL on adipocyte differentiation

**Figure 5:**
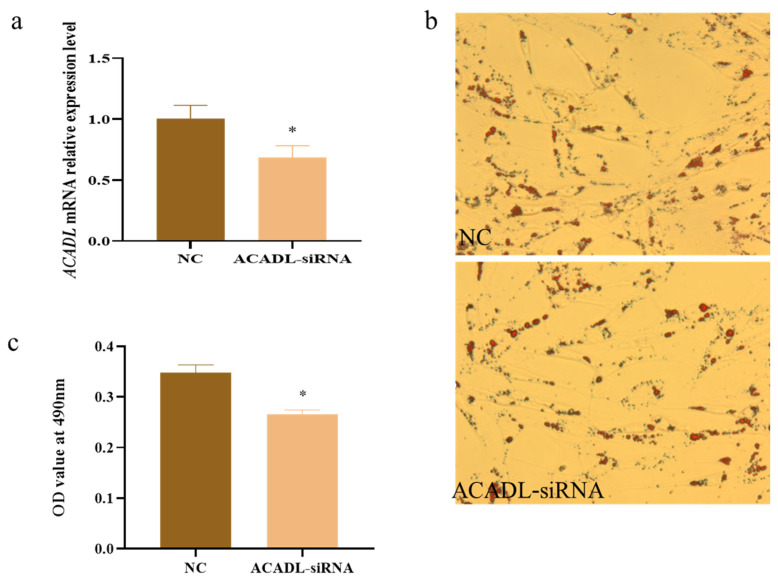
The effect of ACADL interference on goat adipocyte differentiation. (a) Expression efficiency detection after ACADL interference. (b) Morphology observation of oil-red O staining. (c) Oil red staining had showed the OD value at 492 nm during subcutaneous adipocytes differentiation. “*” The difference was significant (p<0.05). ACADL, Acyl-CoA dehydrogenase long chain.

Using UXT as the internal reference gene, ACADL expression was detected by qPCR, and the results showed that the interference efficiency of ACADL was about 40% (Figure 5a). Cells transfected with ACADL siRNA and control were stained with oil red O, and the results showed that adipocytes in the interference treatment group appeared narrow, had lower lipid droplet aggregation, and obtained lower OD values compared to the control group (Figure 5b) (p<0.05). (Figure 5c). In summary, interference with ACADL inhibits the differentiation of subcutaneous adipocytes in goats.

### Effect of ACADL over-expression and ACADL silence on fat-related gene in goat

**Figure 6:**
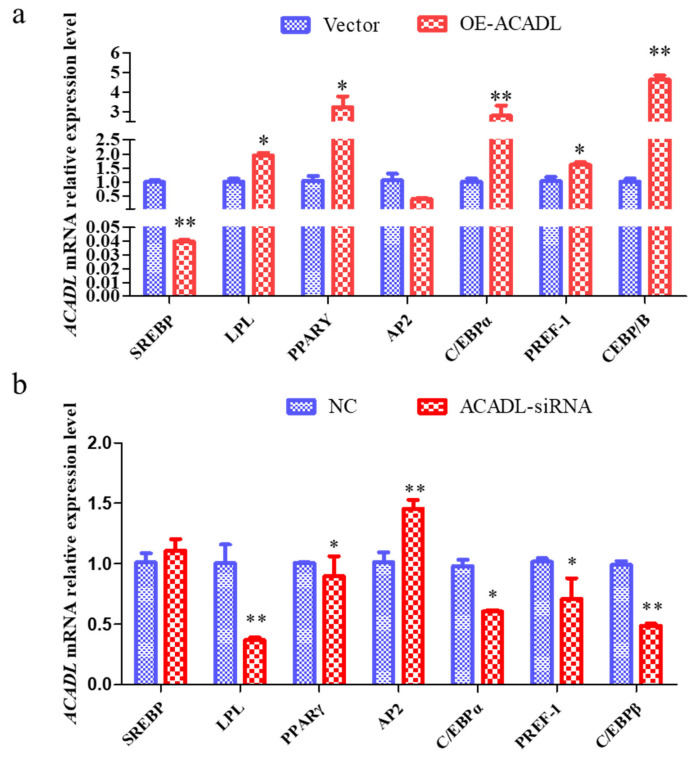
The impact of ACADL on fat-related genes. (a) Effect of goat ACADL overexpression on fat-related gene expression. “**” the difference was extremely significantly compared with control group (p<0.01). “*” The difference was significant (p<0.05). (b) Effect of ACADL-siRNA on fat related gene “**” The difference was extremely significant compared with control group (p<0.01). “*” The difference was significant (p<0.05). ACADL, Acyl-CoA dehydrogenase long chain.

To further elucidate the role of ACADL in goat subcutaneous adipocyte differentiation we examined changes in the expression levels of differentiation marker genes after ACADL overexpression and interference. The expression of *SREBP* gene decreased significantly after overexpression of ACADL but did not change significantly after SI-ACADL. LPL increased significantly after overexpression and decreased significantly after SI-ACADL; PPARγ rises significantly after ACADL overexpression and decreases significantly after SI-ACADL; AP2 did not change significantly after ACADL overexpression, but rose significantly after SI-ACADL; CCAT enhancer binding protein (CEBPα) rises significantly after overexpression and decreases significantly after SI-ACADL; PREF-1 rises significantly after overexpression and decreases significantly after SI-ACADL; CEBPβ rises significantly after ACADL overexpression and decreases very significantly after SI-ACADL (Figure 6a, 6b). In summary, ACADL may be a positive regulator for the differentiation of goat subcutaneous adipocytes.

## DISCUSSION

Long-chain acyl coenzyme A dehydrogenase is the first step in catalytic fatty acid oxidation and plays an important role in long-chain fatty acid oxidative including expression regulation and activity regulation. ACADL is a key factor in multiple metabolism pathways, mice deficient the *ACADL* gene had severe liver and cardiac lipid deposition hypoglycemia, elevated serum free fatty acids and liver insulin resistance due to impaired oxidation of fatty acids [10,11]. The sequence of the CDS region of goat ACADL was successfully cloned in this study, goat ACADL protein plays a biological role as an uncharged, hydrophobic stable protein, mainly localized to the endoplasmic reticulum.

The secondary structure of the ACADL protein is consis tent with the tertiary structure we found, and the extended chain of the ACADL protein’s own structure may be the key to the biological role of ACADL in goats. STRING interactive network analysis discovered that ACADL protein may interact with CPT2, ACOX1, ACAA2, HADHA, HADHB, and ACADVL. CPT2, catalytic fatty acid transport to mitochondria for B oxidation during fat oxidation and CPT2 and ACADL may be positively synergistic [12]. Acyl-Co A oxidase 1 is a rate-limiting enzyme for the first step of dehydrogenation of fatty acid β-oxidation in the peroxisome, which specifically catalyzes the dihydroxylation of long-chain and very long-chain fatty acids to form trans double-bonded double α, β-aleno-lipoyl-CoA. Studies have reported that after overexpression of ACOX1, fat cell differentiation is inhibited [13], besides ACOX1 promotes precursor adipogenesis through both C/EBPα and miR-25-3p regulation [14], so ACOX1 and ACADL may have an antagonistic relationship. ACAA2 inhibits fat differentiation [15], ACAA2 is a key enzyme in the oxidation step of fatty acids, and it has been found that protein lipase receptor activators can increase ACAA2 mRNA expression [16]. Over-expression of ACAA2 promotes precursor fat differentiation [16], HADHA, HADHB possesses an α4/β4 structure and catalyzes the second to fourth reactions of the fatty acid B oxidation cycle and is key partner in ACADL catalytic fatty acid oxidation processes [17,18]. ACADVL fatty acid β the key enzyme in the first step of oxidation, catalyzing the dehydrogenation of 14 to 18 carbons of aleophoyl-CoA, and its defect will lead to the accumulation of carnitine in the long face [19], and it is speculated that ACADVL may have a synergistic relationship with ACADL.

In this study, through nucleic acid alignment and evolu tionary tree construction analysis, it was found that the nucleic acid of the *ACADL* gene in goats had the highest similarity with sheep and was first grouped with Ovis aries in evolutionary tree analysis and was the closest relative, so this is in line with the evolutionary relationship of these species. To obtain the tissue specificity of ACADL in goats, in this experiment, the tissue expression pattern of ACADL in goats was investigated, and found that it was expressed in all of the 10 tissue types examined. ACADL expression was the highest in the sebum fat, followed by the longest dorsal muscle. Our results are similar or different to those reported by other scholars in other species. Zhao et al found ACADL express higher levels in mice, muscle, and liver [20,21]. ACADL widely expressed in muscle, and liver of mice, humans, pigs, etc. Speculation is based on the above report combined with the rest of this experiment, the *ACADL* gene tissue expression is species-and tissue-specific. This study found that ACADL showed a tendency to rise first and then decrease in goat subcutaneous precursor adipocyte differentiation, the expression levels were highest at 24 h. Over-expression of ACADL promotes goat subcutaneous fat differentiation and suppresses its differentiation after interference, ACADL was associated with pig fat deposition and served as a candidate gene by Wang H [20]. Different studies have found that the *ACADL* gene promotes fatty acid metabolism in liver [22]. From these reports and the report of this study, there is possible species specificity of the *ACADL* gene during the regulation of adipocytes in different species. After over-expression of ACADL and interference with ACADL, the effects on the expression of differentiation marker genes, the results showed a relative expression level increase in adipose differentiation marker genes after ACADL over-expression and decreased after interference with ACADL. The main step in energy metabolism is the hydrolyzation of the triacylglycerol protein (TRL), with the released fatty acids which can be used or stored, via the LPL realization in the so-called “binding lipolysis site” of the vascular endothelium [23]. The PPARγ is a “positive regulator” of fat differentiation and inducer of adipogenesis [24]. CEBP/β, an early transcription factor that induces adipogenesis, activates PPARγ by regulating the original transcription of its adjacent promoter. Past studies have reported that C/EBPα has been confirmed as a key gene for fat differentiation. Moreover, it not only regulates AP2 expression, but also promotes the aggregation of lipid droplets, CEBP/α can be combined with CEBP/β, PPARγ to regulate the expression of fatty acid syntheses [25,26]. In this regard, ACADL may promote goat subcutaneous adipocyte differentiation by up regulating the expression of C/EBPα and CEBP/β.

## CONCLUSION

The goat ACADL expressed widely in various goat tissues, among these, the expression was highest in sebum fat (subcutaneous). ACADL expression level was highest in goat preadipocyte when induced differentiation for 24 h. Over-expression of ACADL promotes goat subcutaneous adipocyte differentiation by up regulating the expression of C/EBPα, C/EBPβ, PPARγ, and SREBP. We also confirmed that the expression levels of differentiation markers of adipocyte were decreased after ACADL interference. Our results provide an important theoretical basis for further elucidating the molecular mechanism of the *ACADL* gene in regulating fat deposition in goats.

## Figures and Tables

**Table 1:** Information of primers

Gene	Primer sequence (from 5′to 3′)	TM (°C)	Product length (bp)	Gene bank accession number	Purpose
*ACADL-S*	TTGGGGAAGGTTCAGGGATAGT	60	1,546	XM_018059958	Clone
*ACADL-A*	CGGCAGCCCTCGCTGTCCCAGCC	59	1,546	XM_018059958	Clone
*ACADL-F*	GAATGGGAGAAAGCTGGAGAAG	58	220	XM_018059958	qPCR
*ACADL-R*	GTTTGAAATATAGGGCATGACG	58	220	XM_018059958	qPCR
*C/EBPα-F*	CCGTGGACAAGAACAGCAAC	58	142	XM_018062278.1	qPCR
*C/EBPα-R*	AGGCGGTCATTGTCACTGGT	58	142	XM_018062278.1	qPCR
*C/EBPβ-F*	CAAGAAGACGGTGGACAAGC	66	204	XM_005701796.2	qPCR
*C/EBPβ-R*	AACAAGTTCCGCAGGGTG	66	204	XM_005701796.2	qPCR
*AP2-F*	TGAAGTCACTCCAGATGACAGG	58	143	NM_001285623.1	qPCR
*AP2-R*	TGACACATTCCAGCACCAGC	58	143	NM_001285623.1	qPCR
*LPL-F*	TCCTGGAGTGACGGAATCTGT	60	174	NM_001285607.1	qPCR
*LPL-R*	GACAGCCAGTCCACCACGAT	60	174	NM_001285607.1	qPCR
*PPARγ-F*	AAGCGTCAGGGTTCCACTATG	60	197	NM_001285658.1	qPCR
*PPARγ-R*	GAACCTGATGGCGTTATGAGAC	60	197	NM_001285658.1	qPCR
*SREBP-F*	AAGTGGTGGGCCTCTCTGA	58	127	NM_001285755.1	qPCR
*SREBP-R*	GCAGGGGTTTCTCGGACT	58	127	NM_001285755.1	qPCR
*PREF-1-F*	CGGCTTCATGGATAAGACCT	65	178	KP686197.1	qPCR
*PREF-1-R*	GCCTCGCACTTGTTGAGGAA	65	178	KP686197.1	qPCR

ACADL, Acyl-CoA dehydrogenase long chain; *C/EBPα*, CCAT enhancer binding protein α*; C/EBPβ*, CCAT enhancer binding protein β; *AP2*, APETALA-2-Like transcription factor gene*; LPL*, lipoprotein lipase; *PPARγ*, peroxisome proliferator activated receptor gamma*; SREBP*, sterol regulating element binding protein isoform; *PREF-1*, preadipocyte factor 1; qPCR quantitative real-time polymerase chain reaction.

**Table 2:** The tools for analysis and the results of their analysis are listed in the table

Software or online tools	Analytical content
Primer 5.0	Primer design
ORF Finder	Open reading frame prediction and amino acid sequence translation
ExPASy ProtParam	Analysis of physicochemical properties of protein
ExPASy	Prediction of protein secondary structure
SWISS-MODEL	Prediction of protein tertiary structure
SignalP4.1 Server	Signal peptide analysis
TMHMM	Prediction of transmembrane domain
PSORTII	Subcellular localization
STRING	Protein interaction analysis
MEGA5.0	Construction of phylogenetic tree
Blast	Homology comparison analysis

## SUPPLEMENTARY MATERIAL

**Supplementary Figure S1**. Nucleotide sequence and deduced amino acids of goat ACADL.
