# Corrigendum

**DOI:** 10.5713/ab.22.0308C

**Published:** 2023-06-22

The followings are to be changed in Anim Biosci 2023;36(6):829–839.

**FUNDING**

This work was supported by National Natural Sciences Foundation of China (32072723), Sichuan Science and Technology Program (2022JDTD0030), and Fundamental Research Funds for the Central Universities, Southwest Minzu University (2021057).

