# Supplementary material for: Effect of ACADL on the differentiation of goat subcutaneous adipocyte
Source: Anim Biosci. 2023 Jan 11;36(6):829–39. doi: 10.5713/ab.22.0308 (PMC10164536; doi:10.5713/ab.22.0308)
Supplement: Supplementary file 1 [file ab-22-0308-Supplementary-Fig-1.pdf]

Supplementary

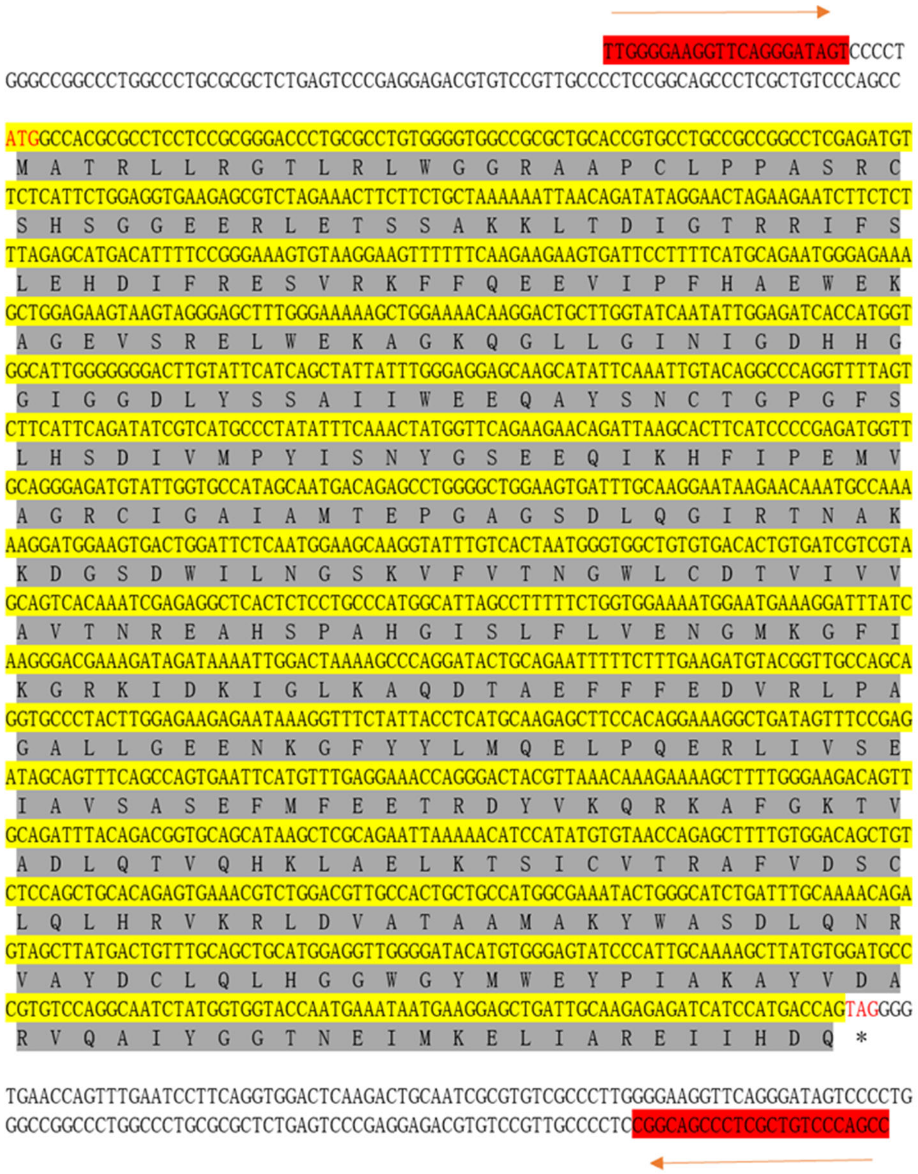

Supplementary Figure S1. Nucleotide sequence and deduced amino acids of goat ACADL. Two blue arrows and red background displayed the position of primers ACADL-S, ACADL-A.
